# Risk of bias tools in systematic reviews of health interventions: an analysis of PROSPERO-registered protocols

**DOI:** 10.1186/s13643-019-1172-8

**Published:** 2019-11-15

**Authors:** Kelly Farrah, Kelsey Young, Matthew C. Tunis, Linlu Zhao

**Affiliations:** Centre for Immunization and Respiratory Infectious Diseases, Public Health Agency of Canada, Ottawa, Canada

**Keywords:** Critical appraisal, Non-randomized studies, PROSPERO, Risk of bias, Systematic reviews

## Abstract

**Background:**

Systematic reviews of health interventions are increasingly incorporating evidence outside of randomized controlled trials (RCT). While non-randomized study (NRS) types may be more prone to bias compared to RCT, the tools used to evaluate risk of bias (RoB) in NRS are less straightforward and no gold standard tool exists. The objective of this study was to evaluate the planned use of RoB tools in systematic reviews of health interventions, specifically for reviews that planned to incorporate evidence from RCT and/or NRS.

**Methods:**

We evaluated a random sample of non-Cochrane protocols for systematic reviews of interventions registered in PROSPERO between January 1 and October 12, 2018. For each protocol, we extracted data on the types of studies to be included (RCT and/or NRS) as well as the name and number of RoB tools planned to be used according to study design. We then conducted a longitudinal analysis of the most commonly reported tools in the random sample. Using keywords and name variants for each tool, we searched PROSPERO records by year since the inception of the database (2011 to December 7, 2018), restricting the keyword search to the “Risk of bias (quality) assessment” field.

**Results:**

In total, 471 randomly sampled PROSPERO protocols from 2018 were included in the analysis. About two-thirds (63%) of these planned to include NRS, while 37% restricted study design to RCT or quasi-RCT. Over half of the protocols that planned to include NRS listed only a single RoB tool, most frequently the Cochrane RoB Tool. The Newcastle-Ottawa Scale and ROBINS-I were the most commonly reported tools for NRS (39% and 33% respectively) for systematic reviews that planned to use multiple RoB tools. Looking at trends over time, the planned use of the Cochrane RoB Tool and ROBINS-I seems to be increasing.

**Conclusions:**

While RoB tool selection for RCT was consistent, with the Cochrane RoB Tool being the most frequently reported in PROSPERO protocols, RoB tools for NRS varied widely. Results suggest a need for more education and awareness on the appropriate use of RoB tools for NRS. Given the heterogeneity of study designs comprising NRS, multiple RoB tools tailored to specific designs may be required.

## Background

With the growing interest in “real-world evidence” obtained from analyzing administrative health data and the development of sophisticated quasi-experimental study designs [1], regulatory agencies [2], and others who systematically review health interventions are increasingly incorporating non-randomized studies (NRS) into their evidence syntheses [3]. As such, methods to appraise the risk of bias, defined as the risk of systematic error in results or inferences [4], of these complex evidence sources are now coming under closer scrutiny. The choice of risk of bias tools (RoB tools) is not straightforward for reviews of NRS, although methodological tools for assessing the risk of bias in randomized controlled trials (RCT) are more well-established, with the Cochrane Collaboration’s RoB Tool [5] now considered the standard [6]. The last two decades have seen a proliferation of tools developed to evaluate the risk of bias in NRS; a 2012 systematic review identified 74 tools developed for quality appraisal, of which risk of bias is a component, of non-experimental studies [7]. However, none of these existing NRS quality appraisal tools are currently accepted as the gold standard [1, 8], and it is unclear which tools are the most rigorous and practical.

Quality appraisal for NRS is complicated by the heterogeneity of this category of study design. Under this umbrella term are a multitude of designs, including experimental studies (e.g., non-randomized controlled clinical trials), quasi-experimental studies (e.g., controlled before-after studies, interrupted time series), and traditional observational studies (e.g., cohort, case-control, cross-sectional studies). NRS may be at higher risk of bias due to confounding compared to RCT [9]; however, a single checklist may not adequately assess the risks particular to the various types of NRS. For example, past studies have found that existing tools are insufficient for the evaluation of the risk of bias in pharmacoepidemiological safety studies [10], natural experimental studies [11], and other quasi-experimental designs [12]. Moreover, if multiple checklists are used in systematic reviews that incorporate multiple study designs, review authors need to consider whether these tools are comparable, particularly in terms of rating evidence within a grading system or when using a cut-off to determine which studies to include in a systematic review or meta-analysis.

Two studies published in 2018 found a wide variation in the use of RoB tools for NRS in published systematic reviews [13, 14]. While the Newcastle-Ottawa Scale was the most frequently used tool for NRS in both studies, it was also not uncommon for systematic reviews to use no RoB tools at all or to inappropriately use tools intended for RCT. Further, Quigley et al. reviewed methodological recommendations from health technology assessment bodies and concluded that there is no consensus on which tool(s) should be the standard of practice for appraising bias in NRS [13].

To our knowledge, no previous study has assessed the use of RoB tools by examining pre-published systematic review protocols, which may provide more detailed methodological information compared to published systematic reviews. Evaluating protocols registered in PROSPERO, an “international prospective register of systematic reviews,” enables us to look forward into the future to anticipate emerging trends in RoB tools, as well as look at historical trends in RoB tool use over time. Given the ongoing development of new RoB tools, certain tools may have fallen out of favor or gained currency over time.

In the present study, we conducted a cross-sectional analysis of systematic review protocols on health interventions registered in PROSPERO to identify which tools were the most commonly cited in 2018 to evaluate the risk of bias of RCT and NRS in systematic reviews. We also conducted a retrospective analysis of trends in the use of these commonly cited RoB tools in protocols of health interventions registered in PROSPERO since database inception (2011). In the absence of a gold standard, identifying the most common tools cited for use would help researchers position their RoB tool selection in the context of their peers. Knowing how RoB tools are applied in practice could also inform future tool development or identify areas where educational interventions on RoB tool use are needed.

## Methods

### Review of 2018 PROSPERO records

#### Data source and sample selection

The search for eligible protocols was conducted using PROSPERO’s database filters for type and method of the review, source of the review, and date of addition to the database [search strategy: (Intervention):RT NOT Cochrane:DB WHERE CD FROM 01/01/2018 TO 12/10/2018]. To be included in this analysis, PROSPERO protocols had to be for systematic reviews of health interventions. We excluded Cochrane review protocols because they were assumed to use Cochrane methodology and RoB tools. Protocols for rapid reviews were excluded as their approach to quality appraisal may be different compared to full systematic reviews. Protocols for overviews of reviews (or “umbrella” reviews), reviews of guidelines, qualitative studies, preclinical studies, and economic evaluations were excluded as the risk of bias assessment for these study designs was outside the scope of this study. Further, we selected protocols from only the most recent year available (2018) in order to determine contemporary practices in the use of RoB tools. Retrieved records were screened by one reviewer (K.F.) for inclusion.

All PROSPERO records that met the date and database review type limits were downloaded on October 12, 2018. There were 4215 eligible protocols registered in PROSPERO from January 1 to October 12, 2018. Of these protocols, 500 (approximately 10% of registered protocols) were randomly selected for practicality, as the aim of this analysis was to identify which RoB tools were the most commonly cited in systematic review protocols in 2018 when this analysis was conducted. A simple random sample was created using the random number generator from RANDOM.org.

#### Data extraction

Data was extracted on the types of studies to be included from each of the selected systematic review protocols. Protocols were then coded as including RCT (including quasi-RCT), NRS (including non-randomized experimental, quasi-experimental, or observational study designs), or both.

Data was also extracted on all of the tools the protocol authors planned to use for risk of bias assessment and, if specified, the study designs that the tools will be used to assess. Since we wanted to understand what RoB tools authors were choosing to use for quality appraisal, we recorded tools according to author intentions and regardless of whether the tools were specifically designed for this purpose. We recorded the systematic review using “suites of tools” in cases where the RoB tool was comprised of separate checklists for different study designs produced by the same organization, but the exact number of checklists to be used was not specified. For example, the Joanna Briggs Institute (JBI) produces a number of tools for appraising various study designs [15]. If authors only refer to JBI tools generally, it is unclear how many tools are being employed. We recorded the review using “multi-design tools” in cases where the RoB tool was designed to assess both RCT and NRS, for example, the Downs and Black checklist [16]. If both RCT and NRS were to be included in the systematic review, we recorded whether the authors planned to use different tools for these designs, or whether they used a single tool for both types. If the authors stated that they were following Cochrane guidelines and only included RCT, we assumed they were using the Cochrane RoB Tool. Data was extracted by one reviewer (K.F.).

### Longitudinal analysis of PROSPERO records

To determine usage trends over time of RoB tools that are in common contemporary use, we searched PROSPERO records for the names of the most frequently cited RoB tools identified in the above cross-sectional analysis to determine how often each tool was mentioned on an annual basis. We assessed annual trends for tools that were named in five or more of the protocols included in the random sample of 2018 PROSPERO records. Tools that were not developed for risk of bias assessment, e.g., reporting guidelines, were excluded. Using keywords and name variants for each tool, we searched PROSPERO records by year since the inception of the database (2011) to December 7, 2018, restricting the keyword search to the “Risk of bias (quality) assessment” field. Searches were limited to protocols for reviews of interventions. Cochrane review protocols were excluded, as it was assumed that they followed the risk of bias procedures outlined in the Cochrane Handbook. The number of records retrieved for each tool per year was recorded. We did not further verify the text of the protocol records. Tools were classified by the types of designs they were intended to assess: RCT only, NRS only, multi-design tools, and suites of tools.

### Statistical analysis

Descriptive statistics were used to summarize the frequency and proportion of the RoB tools in the random sample and year-by-year analysis of PROSPERO records.

## Results

### Included 2018 PROSPERO records

In total, 471 of the 500 PROSPERO protocols from the 2018 random sample were included in the final analysis. Twenty-five protocols were excluded after screening for not meeting pre-specified inclusion criteria and another four protocols were excluded for having unclear information on the types of studies to be included (see flow diagram in Additional file 1). Approximately two-thirds (63%) of the protocols analyzed planned to include NRS, while the remaining 37% of protocols stated that they would limit the analysis to RCT or quasi-RCT. A small proportion of protocols, 2% (10/471), did not anticipate finding any RCT given the nature of the topics, and 1 protocol specifically excluded RCT.

### Risk of bias tools in PROPOSERO-registered protocols

The number of RoB tools listed in protocols according to the types of study designs included is presented in Table 1. Overall, 10% of protocols did not list any specific RoB tool. Over half of the protocols that planned to include NRS in addition to RCT listed only a single RoB tool.

**Table 1:** Number of risk of bias tools listed by study designs included

Number of tools listed	Study designs to be included in systematic review *N* (%)		
	RCT only^†^ *N* = 175	RCT and NRS^‡^ *N* = 296	All protocols *N* = 471
0	15 (8.6)	31 (10.4)	46 (9.8)
1	156 (89.1)	163 (55.1)	319 (67.7)
2	4 (2.3)	77 (26.0)	81 (17.2)
3+	0 (0.0)	9 (3.0)	9 (1.9)
Suite of tools*	0 (0.0)	16 (5.4)	16 (3.4)

*NRS* = non-randomized study, *RCT* = randomized controlled trial

^†^Included quasi-RCT

^‡^Included non-randomized experimental designs, quasi-experimental studies, and observational studies

*Suite of tools: the RoB tool was comprised of separate checklists for different study designs produced by the same organization, but the number of checklists to be used was not specified

As shown in Table 2, in protocols that listed only a single RoB tool, the Cochrane RoB Tool was by far the most commonly cited tool in systematic review protocols including only RCT (85.2%), and to a lesser extent, those including both RCT and NRS (35.6%). The Newcastle-Ottawa Scale and Downs and Black were the next most common tools planned to be used in reviews including both RCT and NRS when a single RoB tool was planned to be used to assess studies. There was a wider variation in the RoB tools listed in reviews including both RCT and NRS compared with RCT only.

**Table 2:** PROSPERO protocols with a single risk of bias tool listed

Study designs to be included in systematic review			
RCT only *N* = 156		RCT and NRS *N* = 163	
Tool	N(%)	Tool	*N* (%)
Cochrane RoB Tool [5]	133 (85.2)	Cochrane RoB Tool [5]	58 (35.6)
PEDro Scale [17]	9 (5.8)	Newcastle-Ottawa Scale [18]	20 (12.3)
Jadad Scale [19]	4 (2.6)	Downs and Black [16]	14 (8.6)
Cochrane RoB 2 Tool [20]	3 (1.9)	ROBINS-I [21]	7 (4.3)
Tool listed < 2 times	7 (4.5)	GRADE approach^*^ [22]	6 (3.7)
		Mixed Methods Assessment Tool [23]	6 (3.7)
		Cochrane Handbook^*^ [24]	5 (3.1)
		PEDro Scale [17]	5 (3.1)
		Jadad Scale [19]	4 (2.5)
		McMaster Critical Review Forum [25]	4 (2.5)
		EPHPP [26]	3 (1.8)
		MINORS [27]	3 (1.8)
		Oxford CEBM Levels of Evidence [28]	3 (1.8)
		Quality Criteria Checklist: Primary Research [29]	3 (1.8)
		NHLBI Pre-Post Quality Appraisal Tool [30]	2 (1.2)
		QUADAS-2 [31]	2 (1.2)
		Tool listed < 2 times	18 (11.0)

*Note*: Tools listed only once were excluded. Unless “2” or “2.0” was specifically stated, it was assumed that the original version of the Cochrane RoB Tool was being referenced

*EPHPP* = Effective Public Health Practice Project tool, *MINORS* = Methodological Index for Non-Randomized Studies, *NHLBI* = National Heart, Lung, and Blood Institute, *NRS* = non-randomized study; Oxford CEBM = Oxford Centre for Evidence-based Medicine Levels of Evidence, *PEDro* = Physiotherapy Evidence Database, *QUADAS* = Quality Assessment of Diagnostic Accuracy Studies, *RCT* = Randomized controlled trial; *RoB* = Risk of bias; *ROBINS-I* = Risk Of Bias In Non-randomized Studies - of Interventions

^+^These are not RoB tools, but were identified as such by protocol authors

Table 3 displays the specific tools mentioned in systematic review protocols that planned to include both RCT and NRS and to use multiple RoB tools. Tools are listed by design, based on the protocol authors’ intentions. When multiple RoB tools were planned to be used in a systematic review, the most commonly listed tool for assessing RCT was the Cochrane RoB Tool. There was limited use of discipline-specific scales, such as the PEDro Scale for assessing studies of physiotherapy interventions. Few protocols specifically mentioned the revised Cochrane RoB 2 Tool. For NRS, the Newcastle-Ottawa Scale and ROBINS-I were the most frequently listed in reviews using multiple RoB tools.

**Table 3:** PROSPERO protocols listing multiple tools by study design

Systematic reviews of both RCT and NRS using design-specific RoB tools *N* = 102			
Tools planned to be used for RCT*		Tools planned to be used for NRS*	
Tool	*N* (%)	Tool	*N* (%)
Cochrane RoB Tool [5]	67 (65.7)	Newcastle-Ottawa Scale [18]	40 (39.2)
JBI Tools [15]	7 (6.9)	ROBINS-I [21]	34 (33.3)
Cochrane RoB 2 Tool [20]	5 (4.9)	JBI Tools [15]	9 (8.8)
Jadad Scale [19]	4 (3.9)	CASP Checklists [32]	8 (7.8)
CASP Checklists [32]	4 (3.9)	NHLBI Tools [30]	5 (4.9)
Cochrane EPOC [33]	2 (2.0)	Cochrane RoB Tool [5]	3 (6.9)
NHLBI Tools [30]	2 (2.0)	Downs and Black [16]	3 (2.9)
PEDro Scale [17]	2 (2.0)	STROBE Statement** [34]	3 (2.9)
Downs and Black [16]	2 (2.0)	Cochrane RoB 2 Tool [20]	2 (2.0)
Tool listed < 2 times	6 (5.9)	MINORS [27]	2 (2.0)
Unclear/none listed	3 (2.9)	NICE Checklists [35]	2 (2.0)
		Tool listed < 2 times	6 (5.9)
		Unclear/none listed	2 (2.0)

*Note*: Tools listed only once were excluded. Unless “2” or “2.0” was specifically stated, it was assumed that the original version of the Cochrane RoB Tool was being referenced

*Some protocols listed multiple tools for RCT or NRS. Proportions reflect planned use of each tool per 102 protocols

**This is not a RoB tool for NRS, but was identified as such by protocol authors

*CASP* = Critical Appraisal Skills Program; EPOC = Effective Practice and Organisation of Care; JBI = Joanna Briggs Institute; MINORS = Methodological Index for Non-Randomized Studies; MMAT = Mixed Methods Assessment Tool; NICE = National Institute for Health and Care Excellence; NHLBI = National Heart, Lung, and Blood Institute; NRS = Non-randomized study; PEDro = Physiotherapy Evidence Database; QUADAS = Quality Assessment of Diagnostic Accuracy Studies; RCT = Randomized controlled trial; RoB = Risk of bias; ROBINS-I = Risk Of Bias In Non-randomized Studies - of Interventions; STROBE = Strengthening The Reporting of Observational Studies in Epidemiology

A full count of all the RoB tools listed in the random sample of protocols is presented in Additional file 2.

### Annual trends in risk of bias tool use in PROSPERO protocols

Fifteen specific RoB tools were listed at least five times in the included 2018 PROSPERO records. Of these, two were excluded (Cochrane Handbook and GRADE approach) because they were guidelines not tools designed for risk of bias assessment. We did not differentiate between the two versions of the Cochrane RoB Tool in the temporal trends analysis, since it was not technically possible in the PROPSERO search interface to search for the term “2.0,” which would be used to identify the revised version of the tool. For ROBINS-I, keywords for the previous version of the tool, “A Cochrane Risk Of Bias Assessment Tool: for Non-Randomized Studies of Interventions” (ACROBAT-NRSI), were also included. A year-by-year search of PROSPERO records was performed on these 12 RoB tools. The full search strategy is provided in Additional file 3.

**Fig. 1:**
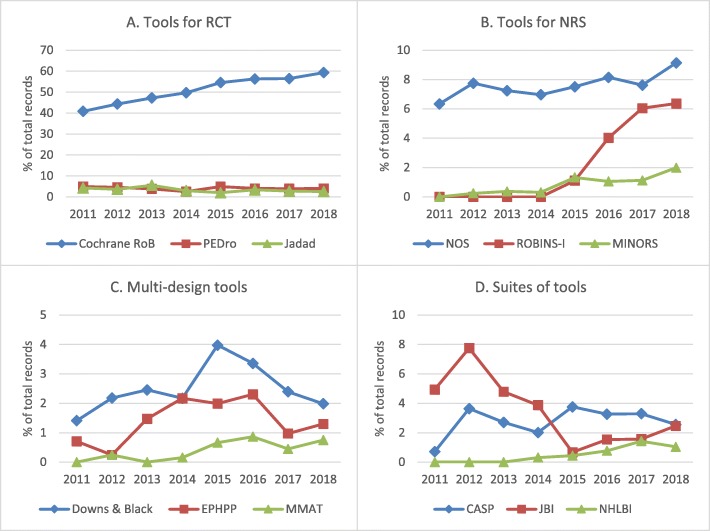
Trends over time for the most frequently cited RoB tools in the included 2018 PROSPERO protocols by type of tool. Percentage of total non-Cochrane systematic review protocols on interventions in PROSPERO, by year, for tools for RCT (**a**), tools for NRS (**b**), multi-design tools (**c**), and suites of tools (**d**). CASP = Critical Appraisal Skills Program; EPHPP = Effective Public Health Practice Project tool; JBI = Joanna Briggs Institute; MINORS = Methodological Index for Non-Randomized Studies; MMAT = Mixed Methods Assessment Tool; NHLBI = National Heart, Lung, and Blood Institute (National Institutes of Health); NOS = Newcastle-Ottawa Scale; NRS = non-randomized studies; PEDro = Physiotherapy Evidence Database; RCT = randomized controlled trial; RoB = risk of bias; ROBINS-I = Risk Of Bias In Non-randomized Studies - of Interventions. Limits: intervention reviews; exclude: Cochrane protocols; restrict to field: assessment of bias

The number of results in the protocols’ “risk of bias” sections for each tool by year is provided in Additional file 4. Of all the RoB tools, the Cochrane RoB Tool had, by far, the highest frequency of planned usage throughout the entire time period, mentioned in over 40% of records every year. Given the generic search terms used for this tool, it is possible these figures are inflated somewhat, but this pattern of use is similarly seen in the random sample of 2018 PROSPERO records. Use of the Cochrane RoB Tool also appears to be increasing over time, rising from 40.8 to 59.3% of protocols from 2011 to December 7, 2018 (see Fig. 1a).

The Newcastle-Ottawa Scale was the next most common tool of the 12 included for analysis and was the most frequently mentioned RoB tool for NRS. Use of the ROBINS-I tools for NRS has increased since it was developed in 2015, rising to 6.4% of the total number of non-Cochrane protocols on health interventions in 2018 (see Fig. 1b).

Multi-design tools were the least commonly mentioned; all three multi-design tools had less than 4% prevalence every year. Of the three multi-design tools searched, the Downs and Black checklist appeared in the highest number of protocols throughout the years reviewed (see Fig. 1c).

Lastly, 3 of the 12 tools were suites of tools. It was not possible to tell using the search results which specific checklist within the suite was being referred to in the PROSPERO protocols. These suites of tools had low frequency of use (< 5% of total records) throughout the entire time period, with the exception of the JBI Critical Appraisal Tools in 2012 (see Fig. 1d).

## Discussion

In this study, two-thirds of PROSPERO protocols on health interventions in the 2018 sample intended to include evidence from NRS in addition to RCT, while the remaining protocols restricted to RCT only. When protocols were restricted to RCT, the choice of RoB tool was highly consistent, with 85.2% planning to use the Cochrane RoB Tool. A few additional protocols (1.9%) planned to use Cochrane RoB 2 Tool, which was first introduced in 2016 as an update to the original Cochrane RoB Tool [20]; however, the uptake of Cochrane RoB 2 Tool may be underestimated, as authors may not have specified the version number in their protocol.

In protocols that intended to include both RCT and NRS, the choice of tools was more heterogeneous, consistent in finding with current opinion that there is no consensus on the preferred tools for evaluating bias in NRS [3, 8, 12, 13]. This finding is also consistent with previous research from Seehra et al., which described quality appraisal tool use in systematic reviews as “varied and inconsistent” [14]. Just over half of protocols including both RCT and NRS listed only one tool for risk of bias assessment, most frequently the Cochrane RoB Tool, which was designed to assess risk of bias in RCT [36]. In a review of 686 systematic reviews, Quigley et al. found that RoB tools designed for RCT were often misapplied to NRS [13]. The choice to use a RoB tool for a study design that it was not intended to be used for might be made for several reasons, such as the convenience of using one tool for multiple study designs, misinformation on appropriate RoB tools, or a lack of a gold standard RoB tool available for NRS. It is also possible that authors had not planned on assessing the quality of NRS. For example, Briere et al. observed that many meta-analyses and health technology assessments using real-world evidence from NRS did not critically appraise these studies [3], and in Deeks et al.’s review of 511 systematic reviews that included NRS, only a third performed quality assessment for NRS [37]. We also found that some protocol authors were not being specific in identifying RoB tools a priori or were inappropriately applying tools to assess risk of bias for NRS. To compound the challenges in appraising the quality of NRS, most of the commonly cited RoB tools for NRS, such as the Newcastle-Ottawa Scale, ROBINS-I, and MINORS, have not been sufficiently validated [13].

When systematic reviews that intended to include NRS planned to use multiple tools to assess risk of bias, the Newcastle-Ottawa Scale was the most commonly listed RoB tool to assess NRS (39%), followed by ROBINS-I (33%). Although some have pointed out that the Newcastle-Ottawa Scale has several weaknesses, including low inter-rater reliability [38] and “uncertain validity” of some items [39], this scale appears to be the most popular choice of all the NRS tools and is considered easy to use [40]. Both Quigley et al. and Seehra et al. also found that the Newcastle-Ottawa Scale was the most frequently used tool to assess risk of bias in NRS. In the trend analysis of commonly listed tools, the Newcastle-Ottawa Scale was the dominant NRS appraisal tool each year, from 2011 to 2018. However, the ROBINS-I tool (previously ACROBAT-NRSI) appears to be gaining in popularity in recent years.

### Limitations

Because this study was conducted using systematic review protocols, we do not know whether the final systematic reviews actually used the tools listed in these protocols. The analysis of PROSPERO protocols for the trend analysis relied on keywords and counts from the search results without further verification in the text of protocols, which may have overestimated the use of certain tools, particularly for Cochrane tools and suites of tools. However, keywords were restricted to the risk of bias section of the registered protocol. As not all systematic reviews are registered prospectively in PROSPERO, results of this study may not be generalizable to the wider body of systematic reviews on health interventions. Authors who are motivated to register systematic reviews in PROSPERO or publish their protocols in peer-reviewed journals, both of which are recommended by the AMSTAR systematic review quality appraisal tool [41], may be more likely to use RoB tools recommended in institutional guidelines, such as the Cochrane Handbook. An additional limitation is that the trend analysis was conducted for only the most commonly cited tools planned for use in systematic reviews in 2018. Therefore, this analysis does not capture complete trends for the planned use of RoB tools over the last 8 years in PROSPERO.

## Conclusions and implications for practice

Results of this analysis emphasize that the Cochrane RoB Tool has become the standard for systematic reviews of RCT. Despite the existence of dozens of tools for assessing NRS, relatively few are commonly used in practice, with the Newcastle-Ottawa Scale and ROBINS-I being the most frequently used. There is also evidence that the Cochrane RoB Tool for RCT may be used inappropriately to assess NRS, indicating a need for more education and awareness on the appropriate use of tools for the quality assessment of non-randomized designs.

With a lack of gold standard for assessing risk of bias in NRS, some have called for the development of an improved tool that could effectively evaluate different kinds of quasi-experimental studies [12]. Others have suggested using different tools based on the types of study designs that are identified by the review [3, 13]. The development of a “meta” quality appraisal tool, such as the one created by Public Health Ontario [42], which recommends particular tools by study design, may be a coherent way to address the lack of guidance on risk of bias assessment for systematic reviews incorporating NRS evidence. Future research should focus on the development and validation of tools for specific NRS designs.

## Supplementary information

**Additional file 1.** Selection of 2018 Sample of PROSPERO Protocols. Sample selection flow diagram.

**Additional file 2.** Risk of Bias Tools Intended to be Used in 2018 PROSPERO Sample. Table with full count of all the risk of bias tools listed in the random sample of protocols.

**Additional file 3.** PROSPERO Annual Trends in Risk of Bias Tools Search Strategy. Full search strategy for 12 commonly used risk of bias tools from 2011 to December 7, 2018 in PROSPERO.

**Additional file 4.** Annual Frequency of Common Tools Listed in PROSPERO Protocol Risk of Bias Section. Full data on number of records that mentioned the 12 commonly used risk of bias tools by year.

## Data Availability

The datasets used in the current study are available from the corresponding author on reasonable request.

## Abbreviations

ACROBAT-NRSI: A Cochrane Risk Of Bias Assessment Tool: for Non-Randomized Studies of Interventions
ADA: American Dietetic Association
AND: Academy of Nutrition and Dietetics
CASP: Critical Appraisal Skills Program
CEBM: Centre for Evidence-based Medicine (Oxford)
EPHPP: Effective Public Health Practice Project
EPOC: Effective Practice and Organisation of Care
JBI: Joanna Briggs Institute
MINORS: Methodological Index for Non-Randomized Studies
MMAT: Mixed Methods Assessment Tool
NHLBI: National Heart, Lung, and Blood Institute
NICE: National Institute for Health and Care Excellence
NRS: Non-randomized study
PEDro: Physiotherapy Evidence Database
QUADAS: Quality Assessment of Diagnostic Accuracy Studies
RCT: Randomized controlled trial
RoB: Risk of bias
ROBINS-I: Risk of Bias in Non-randomized Studies - of Interventions
STROBE: Strengthening the Reporting of Observational Studies in Epidemiology
