# Supplementary material for: Risk of bias tools in systematic reviews of health interventions: an analysis of PROSPERO-registered protocols
Source: Syst Rev. 2019 Nov 15;8:280. doi: 10.1186/s13643-019-1172-8 (PMC6857304; doi:10.1186/s13643-019-1172-8)
Supplement: Supplementary file 1 — Additional file 1. Selection of 2018 Sample of PROSPERO Protocols. Sample selection flow diagram. [file 13643_2019_1172_MOESM1_ESM.docx]

**ADDITIONAL FILE 1: SELECTION OF 2018 SAMPLE OF PROSPERO PROTOCOLS**

4251 interventional non-Cochrane protocols registered in PROSPERO from January 1 to October 12, 2018.

500 protocols randomly selected using random number generator

25 protocols excluded:

-overview of reviews: 8

-SR not on an intervention: 6

-SR of economic evaluations: 3

-SR of preclinical studies: 3

-rapid review: 2

-SR of methodological approaches: 2

-SR of guidelines: 1

475 protocols meeting inclusion criteria

4 protocols excluded for unclear or conflicting statements on which types of study designs were to be included

471 PROSPERO registered protocols included in analysis
