# Supplementary material for: Risk of bias tools in systematic reviews of health interventions: an analysis of PROSPERO-registered protocols
Source: Syst Rev. 2019 Nov 15;8:280. doi: 10.1186/s13643-019-1172-8 (PMC6857304; doi:10.1186/s13643-019-1172-8)
Supplement: Supplementary file 2 — Additional file 2. Risk of Bias Tools Intended to be Used in 2018 PROSPERO Sample. Table with full count of all the risk of bias tools listed in the random sample of protocols. [file 13643_2019_1172_MOESM2_ESM.docx]

**ADDITIONAL FILE 2**: **Risk of Bias Tools Intended to be Used in 2018 PROSPERO Sample**

| **Tools Listed in Risk of Bias Section of Protocol** | **N** | **%** |
| --- | --- | --- |
| Academy of Nutrition & Dietetics/American Dietetic Association Quality Criteria Checklist | 3 | 0.52 |
| Critical Skills Appraisal Program (CASP) Checklists | 9 | 1.57 |
| Clinical Trials Assessment Measure | 1 | 0.17 |
| Cochrane Back Review Group Methods 2009 | 1 | 0.17 |
| Cochrane Effective Practice and Organisation of Care (EPOC) Tools | 3 | 0.52 |
| Cochrane Handbook | 5 | 0.87 |
| Cochrane Risk of Bias Tool | 263 | 45.98 |
| Cochrane Risk of Bias Tool 2.0 | 9 | 1.57 |
| CONSORT (Consolidated Standards Of Reporting Trials) Guidelines | 1 | 0.17 |
| Downs & Black | 18 | 3.15 |
| Effective Public Health Practice Project (EPHPP) tool | 4 | 0.70 |
| GATE (Graphic Appraisal Tool for Epidemiological studies) appraisal checklist | 1 | 0.17 |
| GRADE approach | 7 | 1.22 |
| Health Evidence™ Quality Assessment Tool – Review Articles | 1 | 0.17 |
| Jadad Scale | 15 | 2.62 |
| Joanna Briggs Institute Critical Appraisal Tools | 10 | 1.75 |
| Kratochwill et al. (2010) recommended criteria | 1 | 0.17 |
| Meta Analysis of Statistics Assessment and Review Instrument (MAStARI) | 1 | 0.17 |
| McMaster Quantitative Critical Review Form | 4 | 0.70 |
| MINORS (Methodological Index for Non-Randomized Studies) | 5 | 0.87 |
| Mixed Methods Appraisal Tool (MMAT) | 6 | 0.87 |
| Modified Coleman Methodology Scores | 1 | 0.17 |
| National Health and Medical Research Council (Australian Government) Scale | 1 | 0.17 |
| NICE Checklists (National Institute for Health and Care Excellence) | 2 | 0.35 |
| National Heart, Lung, and Blood Institute (NHLBI) Quality Assessment Tools | 8 | 1.40 |
| Newcastle-Ottawa Scale | 61 | 10.66 |
| OSTEBA statements (Basque Office for Health Technology Assessment) | 1 | 0.17 |
| Oxford Center for Evidence-based Medicine Levels of Evidence (CEBM) | 3 | 0.52 |
| PEDro (Physiotherapy Evidence Database) Scale | 16 | 2.80 |
| QUADAS-2 (Quality Assessment of Diagnostic Accuracy Studies) | 3 | 0.52 |
| QualSyst | 1 | 0.17 |
| QUIPS tool (Quality in Prognostic Studies) | 1 | 0.17 |
| Risk of Bias Assessment tool for Non-randomized Studies (RoBANS) | 1 | 0.17 |
| ROBINS-I (Risk Of Bias In Non-randomized Studies - of Interventions) | 42 | 7.32 |
| ROBIS | 2 | 0.35 |
| Rosendal scale | 1 | 0.17 |
| SIGN (Scottish Intercollegiate Guidelines Network) Checklist for Cohort Studies | 1 | 0.17 |
| STROBE (Strengthening The Reporting of OBservational Studies in Epidemiology) | 4 | 0.70 |
| Tool for the assEssment of Study qualiTy and reporting in EXercise (TESTEX) | 1 | 0.17 |
| US Preventive Services Task Force (USPSTF) criteria | 2 | 0.35 |
| No tool listed/tool listed could not be identified | 52 | 9.23 |
| *Total* | 572 | 100 |

Note: Adapted or modified use of checklists are classified under original name.
