# Supplementary material for: Risk of bias tools in systematic reviews of health interventions: an analysis of PROSPERO-registered protocols
Source: Syst Rev. 2019 Nov 15;8:280. doi: 10.1186/s13643-019-1172-8 (PMC6857304; doi:10.1186/s13643-019-1172-8)
Supplement: Supplementary file 3 — Additional file 3. PROSPERO Annual Trends in Risk of Bias Tools Search Strategy. Full search strategy for 12 commonly used risk of bias tools from 2011 to December 7, 2018 in PROSPERO. [file 13643_2019_1172_MOESM3_ESM.docx]

**ADDITIONAL FILE 3: PROSPERO ANNUAL TRENDS IN RISK OF BIAS TOOLS SEARCH STRATEGY**

**Database:** PROSPERO

**Date of Search:** December 7, 2018

**Date Range:** Inception (2011)- December 7, 2018 (Date added to PROSPERO)

**Filters Applied:** Source of the Review: Exclude Cochrane, Type and Method of the Review: Intervention

**Search Fields**:

BA = Assessment of Bias

RT = Type of Review

DB = Source of the Review

CD = Completion Date (PROSPERO Record)

| **Line** | **Search** | **Hits** |
| --- | --- | --- |
| #1 | (CASP OR critical appraisal skills program):BA AND (Intervention):RT NOT Cochrane:DB WHERE CD FROM 01/01/2018 TO 07/12/2018 | 130 |
| #2 | (CASP OR critical appraisal skills program):BA AND (Intervention):RT NOT Cochrane:DB WHERE CD FROM 01/01/2017 TO 31/12/2017 | 44 |
| #3 | (CASP OR critical appraisal skills program):BA AND (Intervention):RT NOT Cochrane:DB WHERE CD FROM 01/01/2016 TO 31/12/2016 | 34 |
| #4 | (CASP OR critical appraisal skills program):BA AND (Intervention):RT NOT Cochrane:DB WHERE CD FROM 01/01/2015 TO 31/12/2015 | 17 |
| #5 | (CASP OR critical appraisal skills program):BA AND (Intervention):RT NOT Cochrane:DB WHERE CD FROM 01/01/2014 TO 31/12/2014 | 13 |
| #6 | (CASP OR critical appraisal skills program):BA AND (Intervention):RT NOT Cochrane:DB WHERE CD FROM 01/01/2013 TO 31/12/2013 | 22 |
| #7 | (CASP OR critical appraisal skills program):BA AND (Intervention):RT NOT Cochrane:DB WHERE CD FROM 01/01/2012 TO 31/12/2012 | 15 |
| #8 | (CASP OR critical appraisal skills program):BA AND (Intervention):RT NOT Cochrane:DB WHERE CD FROM 01/01/2011 TO 31/12/2011 | 1 |
| #9 | (JBI OR Joanna Briggs):BA AND (Intervention):RT NOT Cochrane:DB WHERE CD FROM 01/01/2018 TO 07/12/2018 | 125 |
| #10 | (JBI OR Joanna Briggs):BA AND (Intervention):RT NOT Cochrane:DB WHERE CD FROM 01/01/2017 TO 31/12/2017 | 21 |
| #11 | (JBI OR Joanna Briggs):BA AND (Intervention):RT NOT Cochrane:DB WHERE CD FROM 01/01/2016 TO 31/12/2016 | 16 |
| #12 | (JBI OR Joanna Briggs):BA AND (Intervention):RT NOT Cochrane:DB WHERE CD FROM 01/01/2015 TO 31/12/2015 | 3 |
| #13 | (JBI OR Joanna Briggs):BA AND (Intervention):RT NOT Cochrane:DB WHERE CD FROM 01/01/2014 TO 31/12/2014 | 25 |
| #14 | (JBI OR Joanna Briggs):BA AND (Intervention):RT NOT Cochrane:DB WHERE CD FROM 01/01/2013 TO 31/12/2013 | 39 |
| #15 | (JBI OR Joanna Briggs):BA AND (Intervention):RT NOT Cochrane:DB WHERE CD FROM 01/01/2012 TO 31/12/2012 | 32 |
| #16 | (JBI OR Joanna Briggs):BA AND (Intervention):RT NOT Cochrane:DB WHERE CD FROM 01/01/2011 TO 31/12/2011 | 7 |
| #17 | (National Institutes of Health OR NIH OR NHLIB OR National Heart Lung):BA AND (Intervention):RT NOT Cochrane:DB WHERE CD FROM 01/01/2018 TO 07/12/2018 | 53 |
| #18 | (National Institutes of Health OR NIH OR NHLIB OR National Heart Lung):BA AND (Intervention):RT NOT Cochrane:DB WHERE CD FROM 01/01/2017 TO 31/12/2017 | 19 |
| #19 | (National Institutes of Health OR NIH OR NHLIB OR National Heart Lung):BA AND (Intervention):RT NOT Cochrane:DB WHERE CD FROM 01/01/2016 TO 31/12/2016 | 8 |
| #20 | (National Institutes of Health OR NIH OR NHLIB OR National Heart Lung):BA AND (Intervention):RT NOT Cochrane:DB WHERE CD FROM 01/01/2015 TO 31/12/2015 | 2 |
| #21 | (National Institutes of Health OR NIH OR NHLIB OR National Heart Lung):BA AND (Intervention):RT NOT Cochrane:DB WHERE CD FROM 01/01/2014 TO 31/12/2014 | 2 |
| #22 | (National Institutes of Health OR NIH OR NHLIB OR National Heart Lung):BA AND (Intervention):RT NOT Cochrane:DB WHERE CD FROM 01/01/2013 TO 31/12/2013 | 0 |
| #23 | (National Institutes of Health OR NIH OR NHLIB OR National Heart Lung):BA AND (Intervention):RT NOT Cochrane:DB WHERE CD FROM 01/01/2012 TO 31/12/2012 | 0 |
| #24 | (National Institutes of Health OR NIH OR NHLIB OR National Heart Lung):BA AND (Intervention):RT NOT Cochrane:DB WHERE CD FROM 01/01/2011 TO 31/12/2011 | 0 |
| #25 | (Cochrane):BA AND (Intervention):RT NOT Cochrane:DB WHERE CD FROM 01/01/2018 TO 07/12/2018 | 3020 |
| #26 | (Cochrane):BA AND (Intervention):RT NOT Cochrane:DB WHERE CD FROM 01/01/2017 TO 31/12/2017 | 746 |
| #27 | (Cochrane):BA AND (Intervention):RT NOT Cochrane:DB WHERE CD FROM 01/01/2016 TO 31/12/2016 | 587 |
| #28 | (Cochrane):BA AND (Intervention):RT NOT Cochrane:DB WHERE CD FROM 01/01/2015 TO 31/12/2015 | 247 |
| #29 | (Cochrane):BA AND (Intervention):RT NOT Cochrane:DB WHERE CD FROM 01/01/2014 TO 31/12/2014 | 321 |
| #30 | (Cochrane):BA AND (Intervention):RT NOT Cochrane:DB WHERE CD FROM 01/01/2013 TO 31/12/2013 | 385 |
| #31 | (Cochrane):BA AND (Intervention):RT NOT Cochrane:DB WHERE CD FROM 01/01/2012 TO 31/12/2012 | 183 |
| #32 | (Cochrane):BA AND (Intervention):RT NOT Cochrane:DB WHERE CD FROM 01/01/2011 TO 31/12/2011 | 58 |
| #33 | (JADAD):BA AND (Intervention):RT NOT Cochrane:DB WHERE CD FROM 01/01/2018 TO 07/12/2018 | 129 |
| #34 | (JADAD):BA AND (Intervention):RT NOT Cochrane:DB WHERE CD FROM 01/01/2017 TO 31/12/2017 | 37 |
| #35 | (JADAD):BA AND (Intervention):RT NOT Cochrane:DB WHERE CD FROM 01/01/2016 TO 31/12/2016 | 35 |
| #36 | (JADAD):BA AND (Intervention):RT NOT Cochrane:DB WHERE CD FROM 01/01/2015 TO 31/12/2015 | 9 |
| #37 | (JADAD):BA AND (Intervention):RT NOT Cochrane:DB WHERE CD FROM 01/01/2014 TO 31/12/2014 | 19 |
| #38 | (JADAD):BA AND (Intervention):RT NOT Cochrane:DB WHERE CD FROM 01/01/2013 TO 31/12/2013 | 45 |
| #39 | (JADAD):BA AND (Intervention):RT NOT Cochrane:DB WHERE CD FROM 01/01/2012 TO 31/12/2012 | 15 |
| #40 | (JADAD):BA AND (Intervention):RT NOT Cochrane:DB WHERE CD FROM 01/01/2011 TO 31/12/2011 | 6 |
| #41 | (PEDro OR Physiotherapy Evidence Database):BA AND (Intervention):RT NOT Cochrane:DB WHERE CD FROM 01/01/2018 TO 07/12/2018 | 206 |
| #42 | (PEDro OR Physiotherapy Evidence Database):BA AND (Intervention):RT NOT Cochrane:DB WHERE CD FROM 01/01/2017 TO 31/12/2017 | 52 |
| #43 | (PEDro OR Physiotherapy Evidence Database):BA AND (Intervention):RT NOT Cochrane:DB WHERE CD FROM 01/01/2016 TO 31/12/2016 | 42 |
| #44 | (PEDro OR Physiotherapy Evidence Database):BA AND (Intervention):RT NOT Cochrane:DB WHERE CD FROM 01/01/2015 TO 31/12/2015 | 22 |
| #45 | (PEDro OR Physiotherapy Evidence Database):BA AND (Intervention):RT NOT Cochrane:DB WHERE CD FROM 01/01/2014 TO 31/12/2014 | 16 |
| #46 | (PEDro OR Physiotherapy Evidence Database):BA AND (Intervention):RT NOT Cochrane:DB WHERE CD FROM 01/01/2013 TO 31/12/2013 | 31 |
| #47 | (PEDro OR Physiotherapy Evidence Database):BA AND (Intervention):RT NOT Cochrane:DB WHERE CD FROM 01/01/2012 TO 31/12/2012 | 19 |
| #48 | (PEDro OR Physiotherapy Evidence Database):BA AND (Intervention):RT NOT Cochrane:DB WHERE CD FROM 01/01/2011 TO 31/12/2011 | 7 |
| #49 | (Newcastle AND Ottawa):BA AND (Intervention):RT NOT Cochrane:DB WHERE CD FROM 01/01/2018 TO 07/12/2018 | 465 |
| #50 | (Newcastle AND Ottawa):BA AND (Intervention):RT NOT Cochrane:DB WHERE CD FROM 01/01/2017 TO 31/12/2017 | 102 |
| #51 | (Newcastle AND Ottawa):BA AND (Intervention):RT NOT Cochrane:DB WHERE CD FROM 01/01/2016 TO 31/12/2016 | 85 |
| #52 | (Newcastle AND Ottawa):BA AND (Intervention):RT NOT Cochrane:DB WHERE CD FROM 01/01/2015 TO 31/12/2015 | 34 |
| #53 | (Newcastle AND Ottawa):BA AND (Intervention):RT NOT Cochrane:DB WHERE CD FROM 01/01/2014 TO 31/12/2014 | 45 |
| #54 | (Newcastle AND Ottawa):BA AND (Intervention):RT NOT Cochrane:DB WHERE CD FROM 01/01/2013 TO 31/12/2013 | 59 |
| #55 | (Newcastle AND Ottawa):BA AND (Intervention):RT NOT Cochrane:DB WHERE CD FROM 01/01/2012 TO 31/12/2012 | 32 |
| #56 | (Newcastle AND Ottawa):BA AND (Intervention):RT NOT Cochrane:DB WHERE CD FROM 01/01/2011 TO 31/12/2011 | 9 |
| #57 | (ROBINS OR ACROBAT):BA AND (Intervention):RT NOT Cochrane:DB WHERE CD FROM 01/01/2018 TO 07/12/2018 | 324 |
| #58 | (ROBINS OR ACROBAT):BA AND (Intervention):RT NOT Cochrane:DB WHERE CD FROM 01/01/2017 TO 31/12/2017 | 81 |
| #59 | (ROBINS OR ACROBAT):BA AND (Intervention):RT NOT Cochrane:DB WHERE CD FROM 01/01/2016 TO 31/12/2016 | 42 |
| #60 | (ROBINS OR ACROBAT):BA AND (Intervention):RT NOT Cochrane:DB WHERE CD FROM 01/01/2015 TO 31/12/2015 | 5 |
| #61 | (ROBINS OR ACROBAT):BA AND (Intervention):RT NOT Cochrane:DB WHERE CD FROM 01/01/2014 TO 31/12/2014 | 0 |
| #62 | (ROBINS OR ACROBAT):BA AND (Intervention):RT NOT Cochrane:DB WHERE CD FROM 01/01/2013 TO 31/12/2013 | 0 |
| #63 | (ROBINS OR ACROBAT):BA AND (Intervention):RT NOT Cochrane:DB WHERE CD FROM 01/01/2012 TO 31/12/2012 | 0 |
| #64 | (ROBINS OR ACROBAT):BA AND (Intervention):RT NOT Cochrane:DB WHERE CD FROM 01/01/2011 TO 31/12/2011 | 0 |
| #65 | (Methodological Index for Non-Randomized Studies OR MINORS) AND (Intervention):RT NOT Cochrane:DB WHERE CD FROM 01/01/2018 TO 07/12/2018 | 101 |
| #66 | (Methodological Index for Non-Randomized Studies OR MINORS) AND (Intervention):RT NOT Cochrane:DB WHERE CD FROM 01/01/2017 TO 31/12/2017 | 15 |
| #67 | (Methodological Index for Non-Randomized Studies OR MINORS) AND (Intervention):RT NOT Cochrane:DB WHERE CD FROM 01/01/2016 TO 31/12/2016 | 11 |
| #68 | (Methodological Index for Non-Randomized Studies OR MINORS) AND (Intervention):RT NOT Cochrane:DB WHERE CD FROM 01/01/2015 TO 31/12/2015 | 6 |
| #69 | (Methodological Index for Non-Randomized Studies OR MINORS) AND (Intervention):RT NOT Cochrane:DB WHERE CD FROM 01/01/2014 TO 31/12/2014 | 2 |
| #70 | (Methodological Index for Non-Randomized Studies OR MINORS) AND (Intervention):RT NOT Cochrane:DB WHERE CD FROM 01/01/2013 TO 31/12/2013 | 3 |
| #71 | (Methodological Index for Non-Randomized Studies OR MINORS) AND (Intervention):RT NOT Cochrane:DB WHERE CD FROM 01/01/2012 TO 31/12/2012 | 1 |
| #72 | (Methodological Index for Non-Randomized Studies) AND (Intervention):RT NOT Cochrane:DB WHERE CD FROM 01/01/2011 TO 31/12/2011 | 0 |
| #73 | (Downs AND Black):BA AND (Intervention):RT NOT Cochrane:DB WHERE CD FROM 01/01/2018 TO 07/12/2018 | 101 |
| #74 | (Downs AND Black):BA AND (Intervention):RT NOT Cochrane:DB WHERE CD FROM 01/01/2017 TO 31/12/2017 | 32 |
| #75 | (Downs AND Black):BA AND (Intervention):RT NOT Cochrane:DB WHERE CD FROM 01/01/2016 TO 31/12/2016 | 35 |
| #76 | (Downs AND Black):BA AND (Intervention):RT NOT Cochrane:DB WHERE CD FROM 01/01/2015 TO 31/12/2015 | 18 |
| #77 | (Downs AND Black):BA AND (Intervention):RT NOT Cochrane:DB WHERE CD FROM 01/01/2014 TO 31/12/2014 | 14 |
| #78 | (Downs AND Black):BA AND (Intervention):RT NOT Cochrane:DB WHERE CD FROM 01/01/2013 TO 31/12/2013 | 20 |
| #79 | (Downs AND Black):BA AND (Intervention):RT NOT Cochrane:DB WHERE CD FROM 01/01/2012 TO 31/12/2012 | 9 |
| #80 | (Downs AND Black):BA AND (Intervention):RT NOT Cochrane:DB WHERE CD FROM 01/01/2011 TO 31/12/2011 | 2 |
| #81 | (Effective Public Health Practice Project OR EPHPP):BA AND (Intervention):RT NOT Cochrane:DB WHERE CD FROM 01/01/2018 TO 07/12/2018 | 66 |
| #82 | (Effective Public Health Practice Project OR EPHPP):BA AND (Intervention):RT NOT Cochrane:DB WHERE CD FROM 01/01/2017 TO 31/12/2017 | 13 |
| #83 | (Effective Public Health Practice Project OR EPHPP):BA AND (Intervention):RT NOT Cochrane:DB WHERE CD FROM 01/01/2016 TO 31/12/2016 | 24 |
| #84 | (Effective Public Health Practice Project OR EPHPP):BA AND (Intervention):RT NOT Cochrane:DB WHERE CD FROM 01/01/2015 TO 31/12/2015 | 9 |
| #85 | (Effective Public Health Practice Project OR EPHPP):BA AND (Intervention):RT NOT Cochrane:DB WHERE CD FROM 01/01/2014 TO 31/12/2014 | 14 |
| #86 | (Effective Public Health Practice Project OR EPHPP):BA AND (Intervention):RT NOT Cochrane:DB WHERE CD FROM 01/01/2013 TO 31/12/2013 | 12 |
| #87 | (Effective Public Health Practice Project OR EPHPP):BA AND (Intervention):RT NOT Cochrane:DB WHERE CD FROM 01/01/2012 TO 31/12/2012 | 1 |
| #88 | (Effective Public Health Practice Project OR EPHPP):BA AND (Intervention):RT NOT Cochrane:DB WHERE CD FROM 01/01/2011 TO 31/12/2011 | 1 |
| #89 | (Mixed Methods Appraisal Tool OR MMAT):BA AND (Intervention):RT NOT Cochrane:DB WHERE CD FROM 01/01/2018 TO 07/12/2018 | 38 |
| #90 | (Mixed Methods Appraisal Tool OR MMAT):BA AND (Intervention):RT NOT Cochrane:DB WHERE CD FROM 01/01/2017 TO 31/12/2017 | 6 |
| #91 | (Mixed Methods Appraisal Tool OR MMAT):BA AND (Intervention):RT NOT Cochrane:DB WHERE CD FROM 01/01/2016 TO 31/12/2016 | 9 |
| #92 | (Mixed Methods Appraisal Tool OR MMAT):BA AND (Intervention):RT NOT Cochrane:DB WHERE CD FROM 01/01/2015 TO 31/12/2015 | 3 |
| #93 | (Mixed Methods Appraisal Tool OR MMAT):BA AND (Intervention):RT NOT Cochrane:DB WHERE CD FROM 01/01/2014 TO 31/12/2014 | 1 |
| #94 | (Mixed Methods Appraisal Tool OR MMAT):BA AND (Intervention):RT NOT Cochrane:DB WHERE CD FROM 01/01/2013 TO 31/12/2013 | 0 |
| #95 | (Mixed Methods Appraisal Tool OR MMAT):BA AND (Intervention):RT NOT Cochrane:DB WHERE CD FROM 01/01/2012 TO 31/12/2012 | 1 |
| #96 | (Mixed Methods Appraisal Tool OR MMAT):BA AND (Intervention):RT NOT Cochrane:DB WHERE CD FROM 01/01/2011 TO 31/12/2011 | 0 |
