# Supplementary material for: Risk of bias tools in systematic reviews of health interventions: an analysis of PROSPERO-registered protocols
Source: Syst Rev. 2019 Nov 15;8:280. doi: 10.1186/s13643-019-1172-8 (PMC6857304; doi:10.1186/s13643-019-1172-8)
Supplement: Supplementary file 4 — Additional file 4. Annual Frequency of Common Tools Listed in PROSPERO Protocol Risk of Bias Section. Full data on number of records that mentioned the 12 commonly used risk of bias tools by year. [file 13643_2019_1172_MOESM4_ESM.docx]

**ADDITIONAL FILE 4: Annual Frequency of Common Tools Listed in PROSPERO Protocol Risk of Bias Section**

|  | **Year** | | | | | | | |  |
| --- | --- | --- | --- | --- | --- | --- | --- | --- | --- |
|  | **2018*** | **2017** | **2016** | **2015** | **2014** | **2013** | **2012** | **2011** | **Total** |
|  | N = 5091 | N = 1339 | N = 1043 | N = 453 | N = 646 | N = 815 | N = 413 | N= 142 | N = 9942 |
| **Tool** | n (%) | n (%) | n (%) | n (%) | n (%) | n (%) | n (%) | n (%) | n (%) |
| **Tools for RCTs** | | | | | | | | | |
| Cochrane RoB Tool | 3020 (59.3) | 756 (56.5) | 587 (56.3) | 247 (54.5) | 321 (49.7) | 385 (47.2) | 183 (44.3) | 58 (40.8) | 5557 (55.9) |
| PEDro Scale | 206 (4.0) | 52 (3.9) | 42 (4.0) | 22 (4.9) | 16 (2.5) | 31 (3.8) | 19 (4.6) | 7 (4.9) | 395 (4.0) |
| Jadad Scale | 129 (2.5) | 37 (3.8) | 35 (3.4) | 9 (2.0) | 19 (2.9) | 45 (5.5) | 15 (3.6) | 6 (4.2) | 295 (3.0) |
| **Tools for NRS** | | | | | | | | | |
| NOS | 465 (9.1) | 102 (7.6) | 85 (8.1) | 34 (7.5) | 45 (7.0) | 59 (7.2) | 32 (7.7) | 9 (6.3) | 831 (8.4) |
| ROBINS-I | 324 (6.4) | 81 (6.0) | 42 (4.0) | 5 (1.1) | 0 (0.0) | 0 (0.0) | 0 (0.0) | 0 (0.0) | 452 (4.5) |
| MINORS | 101 (2.0) | 15 (1.1) | 11 (1.1) | 6 (1.3) | 2 (0.3) | 3 (0.4) | 1 (0.2) | 0 (0.0) | 139 (1.4) |
| **Multi-Design Tools** | | | | | | | | | |
| Downs & Black | 101 (2.0) | 32 (2.4) | 35 (3.4) | 18 (4.0) | 14 (2.2) | 20 (2.5) | 9 (2.2) | 2 (1.4) | 231 (2.3) |
| EPHPP | 66 (1.3) | 13 (1.0) | 24 (2.3) | 9 (2.0) | 14 (2.2) | 12 (1.5) | 1 (0.2) | 1 (0.7) | 140 (1.4) |
| MMAT | 38 (0.7) | 6 (0.4) | 9 (0.9) | 3 (0.7) | 1 (0.2) | 0 (0.0) | 1 (0.2) | 0 (0.0) | 58 (0.6) |
| **Suites of Tools**† | | | | | | | | | |
| CASP | 130 (2.6) | 44 (3.3) | 34 (3.3) | 17 (3.8) | 13 (2.0) | 22 (2.7) | 15 (3.6) | 1 (0.7) | 276 (2.8) |
| JBI | 125 (2.5) | 21 (1.6) | 16 (1.5) | 3 (0.7) | 25 (3.9) | 39 (4.8) | 32 (7.7) | 7 (4.9) | 268 (2.7) |
| NHLBI | 53 (1.0) | 19 (1.4) | 8 (0.8) | 2 (0.4) | 2 (0.4) | 0 (0.0) | 0 (0.0) | 0 (0.0) | 84 (0.8) |

*Up to December 7, 2018

CASP = Critical Appraisal Skills Program; EPHPP = Effective Public Health Practice Project tool; JBI = Joanna Briggs Institute; MINORS = Methodological Index for Non-Randomized Studies; MMAT= Mixed Methods Assessment Tool; NHLBI = National Heart, Lung, and Blood Institute (National Institutes of Health); NOS = Newcastle-Ottawa Scale; NRS = non-randomized studies; PEDro = Physiotherapy Evidence Database; RCT = randomized controlled trials; RoB = Risk of Bias; ROBINS-I = Risk Of Bias In Non-randomized Studies - of Interventions (also searched for ACROBAT-NRSI).

Note: Since protocols can list multiple tools, sum of percentages may exceed 100, or may be under 100 since we only searched specific tools, other tools may have been listed but not captured here.

Limits: Intervention reviews, Exclude Cochrane Protocols, Restrict to field: assessment of bias.

†Suite of tools: risk of bias tools comprised of separate checklists for different study designs produced by the same organization.
